# Anti-tumor effects of retinoids combined with trastuzumab or tamoxifen in breast cancer cells: induction of apoptosis by retinoid/trastuzumab combinations

**DOI:** 10.1186/bcr2625

**Published:** 2010-08-09

**Authors:** Debbie C Koay, Cynthia Zerillo, Murli Narayan, Lyndsay N Harris, Michael P DiGiovanna

**Affiliations:** 1 Department of Internal Medicine (Section of Medical Oncology), Yale Cancer Center and Smilow Cancer Hospital at Yale-New-Haven Hospital, Yale University School of Medicine, 333 Cedar Street, New Haven, CT 06510, USA

## Abstract

**Introduction:**

HER2 and estrogen receptor (ER) are important in breast cancer and are therapeutic targets of trastuzumab (Herceptin) and tamoxifen, respectively. Retinoids inhibit breast cancer growth, and modulate signaling by HER2 and ER. We hypothesized that treatment with retinoids and simultaneous targeting of HER2 and/or ER may have enhanced anti-tumor effects.

**Methods:**

The effects of retinoids combined with trastuzumab or tamoxifen were examined in two human breast cancer cell lines in culture, BT474 and SKBR3. Assays of proliferation, apoptosis, differentiation, cell cycle distribution, and receptor signaling were performed.

**Results:**

In HER2-overexpressing/ER-positive BT474 cells, combining all-*trans *retinoic acid (atRA) with tamoxifen or trastuzumab synergistically inhibited cell growth, and altered cell differentiation and cell cycle. Only atRA/trastuzumab-containing combinations induced apoptosis. BT474 and HER2-overexpressing/ER-negative SKBR3 cells were treated with a panel of retinoids (atRA, 9-*cis*-retinoic acid, 13-*cis*-retinoic acid, or *N*-(4-hydroxyphenyl) retinamide (fenretinide) (4-HPR)) combined with trastuzumab. In BT474 cells, none of the single agents except 4-HPR induced apoptosis, but again combinations of each retinoid with trastuzumab did induce apoptosis. In contrast, the single retinoid agents did cause apoptosis in SKBR3 cells; this was only modestly enhanced by addition of trastuzumab. The retinoid drug combinations altered signaling by HER2 and ER. Retinoids were inactive in trastuzumab-resistant BT474 cells.

**Conclusions:**

Combining retinoids with trastuzumab maximally inhibits cell growth and induces apoptosis in trastuzumab-sensitive cells. Treatment with such combinations may have benefit for breast cancer patients.

## Introduction

HER2 and estrogen receptor (ER) play critical roles in the clinical care of breast cancer patients as both prognostic factors and therapeutic targets. Approximately 25% of invasive breast tumors have overexpression/amplification of HER2, which is an adverse prognostic factor [1,2]. Trastuzumab (Herceptin), a humanized monoclonal antibody against the extracellular domain of HER2 [3-5], has shown significant therapeutic benefit in the treatment of patients with HER2-overexpressing breast cancer [6-20]. Approximately 60% of primary breast cancers are ER-positive [21,22]. The selective ER modulator tamoxifen is highly effective standard therapy for all stages of endocrine-responsive breast cancer.

Retinoids inhibit growth of breast cancer cell lines in culture and inhibit breast tumor growth in animal models. Retinoid signals are mediated through the retinoic acid receptors (RARs) and the retinoid X receptors (RXRs), with each family represented by three distinct receptor genes designated α, β, and γ [23-25]. All-*trans *retinoic acid (atRA) preferentially binds RARs but not RXRs [23-25]; however, atRA can be converted intracellularly to 9-*cis*-retinoic acid (9-*cis*-RA), an RXR ligand [26]. 9-*cis*-RA and 13-*cis*-RA bind both RARs and RXRs. *N*-(4-hydroxyphenyl) retinamide (4-HPR, fenretinide) is a synthetic analog of atRA [23-25] that has also shown anti-tumor activity, but may have differing mechanisms of action. Following stimulation by retinoids, RAR-RXR heterodimers and RXR-RXR homodimers can form [23-25]. The receptor dimers bind to retinoic acid response elements or retinoid X response elements in the promoter sequences of target genes, and they modulate gene transcription [23-25].

Effects of retinoids on signaling by ER and HER2 have been reported. Inhibition of breast tumor cell growth by retinoids is greater for ER-positive cells than ER-negative cells [27], which may be partly related to alterations in retinoid metabolism [28]. Some studies have found that RA increased the amount of ER in MCF7 breast cancer cells [29], although others reported that RA downregulated ER in this cell line [30]. Regardless, activated RARs have been observed to exert anti-estrogenic effects by directly or indirectly impairing binding of ER to estrogen response elements (EREs) [31]. Conversely, the N-terminal region of ERα modulates the transcriptional activity of RAR [32].

Both RARα and RARβ have been implicated in the anti-proliferative effects of retinoids against breast cancer. RARα expression is correlated positively with ER and with RA sensitivity, and is inducible by estrogen [27]. RARβ has been ascribed tumor suppressor-type activity and is often down regulated in breast cancer; it is inducible by atRA, and inducibility correlates with atRA sensitivity [27]. In both ER-positive T47D cells and ER-negative SKBR3 cells, some evidence suggests that RARα is the receptor solely sufficient for the growth inhibition, cell cycle arrest, apoptosis, and modulation of RAR levels [33].

Inhibition of breast cancer cell growth by atRA and 4-HPR has been associated with downregulation of HER2; while atRA induced morphologic changes consistent with differentiation, 4-HPR induced those of apoptosis [34]. Another study demonstrated that RA can induce differentiation of cultured breast tumor cells, and this was again associated with reduction in cell surface HER2 [35]. atRA and 9-*cis*-RA caused decreases in HER2 and HER3, and inhibited SKBR3 cell growth with cell cycle arrest and induction of apoptosis [36], and the retinoids downregulated HER4 in T47D cells [37]; atRA also downregulated HER2 and HER3 in MCF cells [38]. 4-HPR was reported in another study to downregulate both HER2 and the epidermal growth factor receptor (EGFR, HER1) [39]. In contrast, stimulation of SKBR3 cells with epidermal growth factor or heregulin β1 (HRGβ1) upregulates RARα expression [40], yet resistance to atRA-induced growth inhibition has been reported for HER2-overexpressing breast cancer cells (either HER2-transfected MCF7 cells or naturally overexpressing BT474 or MDA-MB-453 cells); pretreatment for several days with trastuzumab could sensitize these latter two cell lines to inhibition by atRA [41,42]. Trastuzumab treatment increased RA response element binding activity measured by electrophoretic mobility shift assay in a HER2-overexpressing cell line [41]. Co-amplification of RARα with HER2 has been reported in human breast tumors [43]. Finally, retinoids have been found to delay the onset of mammary tumors in HER2 transgenic mice [44,45], and a selective ER modulator/rexinoid combination was synergistic in the prevention or treatment of such tumors, despite the ER-negative status of such tumors [46].

Given the known interactions of retinoids with ER and HER2, we hypothesize that treatment with retinoids and simultaneous targeting of HER2 and/or ER may be a fruitful approach to treating breast cancer. As models, we have used ER-positive (BT474) and ER-negative (SKBR3) HER2-overexpressing human breast cancer cell lines. In the present article we examine the effects of various retinoids (atRA, 9-*cis*-RA, 13-*cis*-RA, and 4-HPR), trastuzumab, tamoxifen, or combinations of these drugs on proliferation, cell cycle, differentiation, and apoptosis in BT474 and SKBR3 cells. Since retinoids, trastuzumab, and tamoxifen are individually agents that possess anti-tumor activity toward breast cancer, combinations of these drugs may translate into improved therapy for breast cancer patients. We report synergistic inhibition of cell proliferation for combinations of these drugs, but apoptosis-inducing activity only of the retinoid/trastuzumab combinations.

## Materials and methods

### Drugs

Trastuzumab (Herceptin) was a gift from Genentech (South San Francisco, CA, USA), supplied as a stock solution in PBS. Tamoxifen citrate, atRA, 9-*cis*-RA, 13-*cis*-RA, and 4-HPR were purchased from Sigma-Aldrich (St Louis, MO, USA); stock solutions of these drugs were prepared in 100% ethanol (EtOH) and were kept light-protected. HRGβ1 was purchased from R&D Systems (Minneapolis, MN, USA) and was reconstituted in PBS.

### Cell culture

BT474 cells [47] and SKBR3 cells were obtained from American Type Culture Collection (Manassas, VA, USA). BT474 cells - which are ER-positive, estrogen dependent, and HER2 overexpressing [48-50] - were cultured in RPMI 1640 medium (GIBCO, Grand Island, NY, USA) supplemented with 10% heat-inactivated FBS (GIBCO), 2 mM l-glutamine (GIBCO), 10 μg/ml bovine insulin (Sigma-Aldrich), and penicillin/streptomycin (GIBCO) at 37°C in a 5% carbon dioxide/95% air-humidified incubator. SKBR3 cells - which are ER-negative and HER2 overexpressing - were cultured in McCoy's 5A medium with l-glutamine (GIBCO) supplemented with 15% FBS (GIBCO) and penicillin/streptomycin (GIBCO).

### WST-1 colorimetric cell proliferation assay

BT474 cells or SKBR3 cells were seeded in 96-well plates at 10,000 or 5,000 cells per well, respectively. On the following day, the cells were treated with vehicle (EtOH + PBS) or drug(s). In each independent experiment, eight replicate wells of cells were used for each treatment. On day 6 following treatment, the WST-1 proliferation assay was performed according to the protocol provided by the manufacturer (Roche Applied Science, Indianapolis IN, USA). Results are expressed as a percentage of control (vehicle-treated cells).

### Analysis of drug interactions

Drug interaction results from the WST-1 proliferation assay were examined by the method of Chou and Talalay [51] using the commercially available software CalcuSyn [52,53] (Biosoft, Ferguson, MO, USA). The Chou-Talalay method is based on the median-effect equation for the dose-effect relationship:

$$f_{\text{a}}/f_{\text{u}}=\left(D/D_{\text{m}}\right)m$$

which can be linearly transformed as:

$$\text{log }\left(f_{\text{a}}/f_{\text{u}}\right)=m\text{ log}\left(D\right)–m\text{ log }\left(D_{\text{m}}\right)$$

where *f*_a _is the fraction affected, *f*_u _is the fraction unaffected, *D *is the dose, *D*_m _is the dose that produces a median effect (IC50), and *m *is the coefficient signifying the sigmoidicity of the curve (or the slope in a linear transformation).

For examining the effect of multiple drugs, a combination index (CI) is calculated based on the doses that have equivalent effects. The formula used for calculating the CI of two drugs is:

CI=(D1c/D1)+(D2c/D2)

and the formula used for determining the CI of three drugs is:

CI=(D1c/D1)+(D2c/D2)+(D3c/D3)

where D1, D2, and D3 are the doses for each drug alone that inhibit a certain percentage, and D1c, D2c, and D3c are the doses for each drug in a combination that inhibit the same percentage. The CI is a quantitative measurement of the degree of interaction between two or more drugs: CI < 1 indicates synergism between the drugs, CI = 1 indicates additivity, and CI > 1 denotes antagonism.

### Analysis of cell cycle and detection of apoptosis by determination of sub-G_1 _DNA peak

BT474 cells or SKBR3 cells were seeded in 25 cm^2 ^flasks at 1 × 10^6 ^or 0.5 × 10^6 ^cells per flask, respectively. On the following day, the cells were treated with vehicle (EtOH + PBS) or drug(s). At designated time points, floating cells in the growth media were collected, and adherent cells were trypsinized and collected. The pooled floating and adherent cells were washed twice with cold PBS. The washed cells were then resuspended in 2 ml of cold PBS, fixed by three stepwise additions of 2 ml each of cold 95% EtOH, and were stored at 4°C. For analysis of cell cycle and sub-G_1 _DNA peak [54], the fixed cells were pelleted by centrifugation, incubated with 1 mg/ml ribonuclease A (Sigma-Aldrich) in PBS at 37°C for 30 minutes, and stained on ice with 50 μg/ml propidium iodide (Sigma-Aldrich) in PBS for 1 hour. The cell cycle distribution (percentages of cells in the G_0_/G_1_, S, and G_2_/M phases) and the percentage of cells in the sub-G_1 _DNA peak were determined by flow cytometry.

### Detection of apoptosis by annexin V assay

BT474 cells were seeded in 25 cm^2 ^flasks at 10^6 ^cells per flask. On the following day, cells were treated with drugs or vehicle. At designated time points, floating cells in the growth media were collected, and adherent cells were trypsinized and collected. The pooled floating and adherent cells were washed with cold PBS. The cells were then stained using Vybrant Apoptosis Assay Kit #2 (Molecular Probes, Eugene, OR, USA). Briefly, the cells were incubated with Alexa Fluor 488 annexin V and propidium iodide in 1× Annexin-Binding Buffer (provided with the kit) for 15 minutes at room temperature. The percentages of annexin V-positive and propidium iodide-positive cells were determined by flow cytometry.

### Detection of differentiation by Nile red staining of neutral lipids

BT474 cells or SKBR3 cells were seeded in 25 cm^2 ^flasks at 1 × 10^6 ^or 0.5 × 10^6 ^cells per flask, respectively. On the following day, the cells were treated with vehicle (EtOH + PBS), or drug(s). On day 6 following treatment, the cells were collected by trypsinization, washed with PBS, and stained with 100 ng/ml Nile red fluorescent dye (Sigma-Aldrich) in PBS for 5 minutes at room temperature. The stained cells were then washed twice with PBS, resuspended in PBS, and analyzed by flow cytometry [55].

### Immunoblot experiments

Immunoblotting was performed on cell extracts by standard techniques using the following antibodies. Antibodies to RARα (sc-551), RARβ (sc-552), RXRα (sc-553), RXRβ (sc-742) and HER2 (sc-284) were from Santa Cruz Biotechnology (Santa Cruz, CA, USA). Antibodies to AKT, phospho-AKT, mitogen-activated protein kinase (MAPK) and phospho-MAPK were from Cell Signaling Technology (Beverly, MA, USA). Antibody to phospho-HER2 (Tyr-1248) (c-erbB-2/HER-2/*neu *phospho-specific Ab-18) was from NeoMarkers (Fremont, CA, USA) and antibody to actin was from Sigma (St Louis, MO, USA).

### Measurement of estrogen receptor transcriptional activity by dual luciferase reporter assay

BT474 cells were seeded in six-well plates at 10^6 ^cells per well. On the following day, the cells were transiently co-transfected with Renilla luciferase reporter vector plasmid pRL-CMV (Promega, Madison, WI, USA) (a control to normalize for transfection efficiency) and a plasmid containing three consensus EREs fused to a firefly luciferase reporter vector (3 × ERE-TATA-Luc) described previously [56,57] using the TransFast transfection reagent (Promega) according to the manufacturer's protocol. Briefly, each well of cells was incubated at 37°C for 30 minutes in serum-free RPMI media containing 1,000 ng 3 × ERE-TATA-Luc reporter vector, 20 ng Renilla luciferase reporter vector pRL-CMV, and TransFast transfection reagent. Following incubation, the transfection mixture was removed, and normal growth media (including 10% FBS without charcoal stripping) was added to the cells, followed immediately by addition of experimental drugs. Drug treatment was with vehicle (EtOH + PBS), 1 μM Faslodex, 1 μM atRA, 1 μg/ml trastuzumab, 1 μM tamoxifen, or the various drug combinations at the same concentrations. Two days following transfection and treatment, the dual luciferase reporter assay was performed using the Promega Dual Luciferase Reporter Assay System according to the manufacturer's protocol. The firefly luciferase activities of the treated cells were normalized to their Renilla luciferase activities and are expressed as a percentage of activity of untreated cells.

### Production of trastuzumab-resistant BT474 cells

Trastuzumab-resistant BT474 cells were selected as described [58] by long-term culture in media containing trastuzumab at 100 μg/ml. Cells were maintained in the same trastuzumab-containing media unless otherwise indicated.

## Results

### Analysis of interactions between atRA, trastuzumab, and tamoxifen on cell proliferation

ER-positive/HER2-overexpressing BT474 cells were treated with atRA, trastuzumab or tamoxifen at a range of doses (0.2 to 10 μM for atRA and tamoxifen, and 0.2 to 10 μg/ml for trastuzumab) and with various combinations of the three drugs at fixed dose ratios. On day 6 following treatment, the effects of the single agents and drug combinations on BT474 cell growth were examined by the WST-1 proliferation assay. The results from WST-1 assays were expressed as a percentage of control growth, and drug interactions were analyzed by the Chou-Talalay method.

Each single agent demonstrated dose-dependent inhibition of cell proliferation (Tables 1, 2, 3 and 4). All combinations of atRA/trastuzumab, of atRA/tamoxifen, of trastuzumab/tamoxifen (except a dose of 0.2 μM), and of the atRA/trastuzumab/tamoxifen triple combination examined were synergistic (most were strongly synergistic with CI < 0.3 or very strongly synergistic with CI < 0.1) (Tables 1, 2, 3 and 4).

**Table 1:** WST-1 proliferation assay for BT474 cells treated with atRA and trastuzumab

	Fraction affected			
		
Dose	atRA	Trastuzumab	atRA + Tzmab	Combination index
0.2	0.17 ± 0.052	0.26 ± 0.009	0.56 ± 0.021	0.19 ± 0.057
0.4	0.28 ± 0.056	0.51 ± 0.006	0.79 ± 0.037	0.01 ± 0.003
0.6	0.33 ± 0.058	0.58 ± 0.012	0.83 ± 0.049	0.01 ± 0.007
1	0.46 ± 0.046	0.62 ± 0.026	0.86 ± 0.042	0.01 ± 0.005
5	0.52 ± 0.061	0.64 ± 0.023	0.85 ± 0.052	0.07 ± 0.046
10	0.55 ± 0.058	0.66 ± 0.030	0.87 ± 0.048	0.10 ± 0.067

Doses of all-*trans *retinoic acid (atRA) and trastuzumab (Tzmab) in μM and μg/ml, respectively. Each value is the mean of three independent experiments (with eight replicate wells for each treatment) ± standard error.

**Table 2:** WST-1 proliferation assay for BT474 cells treated with atRA and tamoxifen

	Fraction affected			
		
Dose	atRA	Tamoxifen	atRA + Tam	Combination index
0.2	0.21 ± 0.012	0.16 ± 0.059	0.33 ± 0.015	0.53 ± 0.099
0.4	0.26 ± 0.075	0.23 ± 0.049	0.46 ± 0.020	0.27 ± 0.066
0.6	0.37 ± 0.026	0.29 ± 0.075	0.51 ± 0.035	0.24 ± 0.062
1	0.47 ± 0.043	0.33 ± 0.032	0.60 ± 0.027	0.16 ± 0.048
5	0.51 ± 0.067	0.42 ± 0.029	0.62 ± 0.050	0.64 ± 0.072
10	0.53 ± 0.071	0.59 ± 0.045	0.69 ± 0.050	0.61 ± 0.106

Doses of all-*trans *retinoic acid (atRA) and tamoxifen (Tam) in μM. Each value is the mean of three independent experiments (with eight replicate wells for each treatment) ± standard error.

**Table 3:** WST-1 proliferation assay for BT474 cells treated with trastuzumab and tamoxifen

	Fraction affected			
		
Dose	Trastuzumab	Tamoxifen	Tzmab + Tam	Combination index
0.2	0.27 ± 0.006	0.20 ± 0.028	0.39 ± 0.020	1.27 ± 0.212
0.4	0.49 ± 0.009	0.21 ± 0.022	0.60 ± 0.038	0.26 ± 0.072
0.6	0.58 ± 0.012	0.34 ± 0.040	0.69 ± 0.026	0.13 ± 0.027
1	0.64 ± 0.017	0.36 ± 0.021	0.75 ± 0.021	0.10 ± 0.017
5	0.66 ± 0.012	0.48 ± 0.015	0.82 ± 0.022	0.19 ± 0.047
10	0.68 ± 0.012	0.64 ± 0.012	0.90 ± 0.012	0.07 ± 0.016

Doses of trastuzumab (Tzmab) and tamoxifen (Tam) in μg/ml and μM, respectively. Each value is the mean of three independent experiments (with eight replicate wells for each treatment) ± standard error.

**Table 4:** WST-1 proliferation assay for BT474 cells treated with atRA, trastuzumab, and tamoxifen

	Fraction affected				
		
Dose	atRA	Trastuzumab	Tamoxifen	atRA + Tzmab + Tam	Combination index
0.2	0.20 ± 0.028	0.24 ± 0.017	0.10 ± 0.063	0.58 ± 0.017	0.15 ± 0.014
0.4	0.29 ± 0.041	0.48 ± 0.032	0.15 ± 0.036	0.84 ± 0.007	0.01 ± 0.001
0.6	0.37 ± 0.023	0.58 ± 0.012	0.25 ± 0.087	0.89 ± 0.010	0.01 ± 0.001
1	0.46 ± 0.046	0.63 ± 0.023	0.29 ± 0.055	0.90 ± 0.006	0.01 ± 0.003
5	0.55 ± 0.038	0.65 ± 0.015	0.37 ± 0.055	0.89 ± 0.009	0.06 ± 0.011
10	0.57 ± 0.041	0.66 ± 0.030	0.59 ± 0.048	0.90 ± 0.003	0.09 ± 0.020

Doses of all-*trans *retinoic acid (atRA), trastuzumab (Tzmab), and tamoxifen (Tam) in μM, μg/ml, and μM, respectively. Each value is the mean of three independent experiments (with eight replicate wells for each treatment) ± standard error.

### Analysis of cell cycle following drug treatment

**Figure 1:**
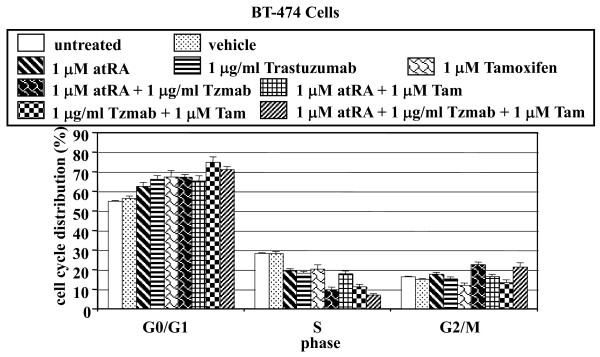
**Cell cycle analysis**. BT474 cells were either untreated or treated with vehicle (ethanol + PBS), 1 μM all-*trans *retinoic acid (atRA), 1 μg/ml trastuzumab, 1 μM tamoxifen, 1 μM atRA + 1 μg/ml trastuzumab (Tzmab), 1 μM atRA + 1 μM tamoxifen (Tam), 1 μg/ml trastuzumab (Tzmab) + 1 μM tamoxifen (Tam), or 1 μM atRA + 1 μg/ml trastuzumab (Tzmab) + 1 μM tamoxifen (Tam). On day 2 following treatment, both floating and adherent cells were collected and fixed. The percentages of cells in the G_0_/G_1_, S, and G_2_/M phases of the cell cycle were determined by flow cytometric analyses. Results are mean of three independent experiments ± standard error.

The cell cycle distribution of BT474 cells was analyzed following treatment with single agents or various combinations of atRA, tamoxifen or trastuzumab. Each drug individually is known to cause G_1 _cell cycle accumulation. Compared with untreated cells and vehicle-treated cells, single agents and various combinations of the three drugs led to an enhanced accumulation of cells in the G_0_/G_1 _phase coupled with a reduction of cells in the S phase of cell cycle (Figure 1). In general, the drug combinations produced lower percentage of cells in the S phase than did single agents.

### Analysis of differentiation following drug treatment

**Figure 2:**
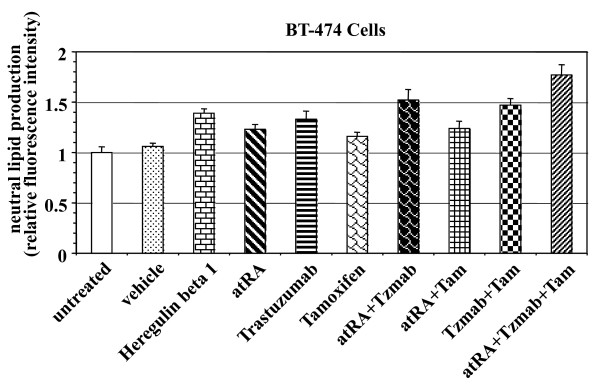
**Differentiation of BT474 cells as determined by Nile red staining of neutral lipids**. BT474 cells were either untreated or treated with vehicle (ethanol + PBS), 50 ng/ml heregulin β1, 1 μM all-*trans *retinoic acid (atRA), 1 μg/ml trastuzumab, 1 μM tamoxifen, 1 μM atRA + 1 μg/ml trastuzumab (Tzmab), 1 μM atRA + 1 μM tamoxifen (Tam), 1 μg/ml trastuzumab (Tzmab) + 1 μM tamoxifen (Tam), or 1 μM atRA + 1 μg/ml trastuzumab (Tzmab) + 1 μM tamoxifen (Tam). On day 6 following treatment, the cells were collected and stained with the fluorescent dye Nile red. The fluorescence intensities of the cells were analyzed by flow cytometry and expressed relative to the intensity of the untreated cells. Results are mean of eight independent experiments ± standard error.

Differentiation of BT474 cells was determined by Nile red fluorescent dye staining of neutral lipids on day 6 following treatment with HRGβ1 (positive control), single agents, or various combinations of atRA, tamoxifen or trastuzumab. Compared with untreated and vehicle-treated cells, HRGβ1, single agents, or various drug combinations led to an increase in neutral lipid production (Figure 2). Treatment with the atRA/trastuzumab, trastuzumab/tamoxifen, and atRA/trastuzumab/tamoxifen combinations resulted in greater neutral lipid production than the respective single agents alone (*P *< 0.05) (Figure 2). The triple combination induced the greatest degree of differentiation (*P *< 0.05) (Figure 2).

### Analysis of apoptosis following drug treatment

**Figure 3:**
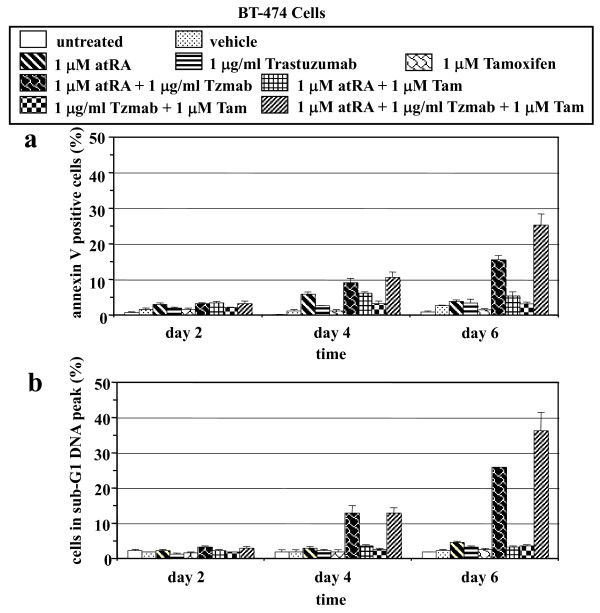
**Detection of apoptotic BT474 cells by annexin V staining and sub-G**_**1 **_**DNA peak analysis**. BT-474 cells were either untreated or treated with vehicle (ethanol + PBS), 1 μM all-*trans *retinoic acid (atRA), 1 μg/ml trastuzumab, 1 μM tamoxifen, 1 μM atRA + 1 μg/ml trastuzumab (Tzmab), 1 μM atRA + 1 μM tamoxifen (Tam), 1 μg/ml trastuzumab (Tzmab) +1 μM tamoxifen (Tam), or 1 μM atRA + 1 μg/ml trastuzumab (Tzmab) + 1 μM tamoxifen (Tam). On days 2, 4, and 6 following treatment, both floating and adherent cells were collected and examined by **(a) **annexin V staining or **(b) **sub-G_1 _DNA peak analysis. Percentages of (a) annexin V-positive cells or (b) cells in the sub-G_1 _DNA peak were determined by flow cytometry. Results are mean of three independent experiments ± standard error.

To detect apoptotic cells, both floating and adherent BT474 cells were examined by annexin V staining and sub-G_1 _DNA peak analysis following treatment with single agents or various combinations of the three drugs. Both annexin V staining and sub-G_1 _DNA peak analysis demonstrate that only the atRA/trastuzumab and atRA/trastuzumab/tamoxifen combinations induced apoptosis (Figure 3). The atRA/trastuzumab combination resulted in 3%, 9%, and 16% annexin V-positive cells (Figure 3a), and in 3%, 13%, and 26% cells in the sub-G_1 _DNA peak (Figure 3b) on days 2, 4, and 6, respectively. The atRA/trastuzumab/tamoxifen combination induced the greatest percentage of apoptotic cells (3%, 11%, and 25% annexin V-positive cells, and 3%, 13%, and 36% cells in the sub-G_1 _DNA peak on days 2, 4, and 6, respectively) (Figure 3). Therefore, while neither the single agents nor the atRA/tamoxifen or trastuzumab/tamoxifen combinations induced apoptosis, the atRA/trastuzumab and atRA/trastuzumab/tamoxifen combinations did result in apoptosis.

Given the unique ability of the atRA/trastuzumab combination to induce apoptosis in ER-positive BT474 cells, and the known interaction between retinoids and ER signaling discussed above, it was of interest to extend these experiments to ER-negative/HER2-overexpressing cells. In addition, it was of interest to compare the effects of other retinoids to those of atRA.

### Effect of other retinoids with trastuzumab on cell proliferation of ER-positive and ER-negative cells

**Figure 4:**
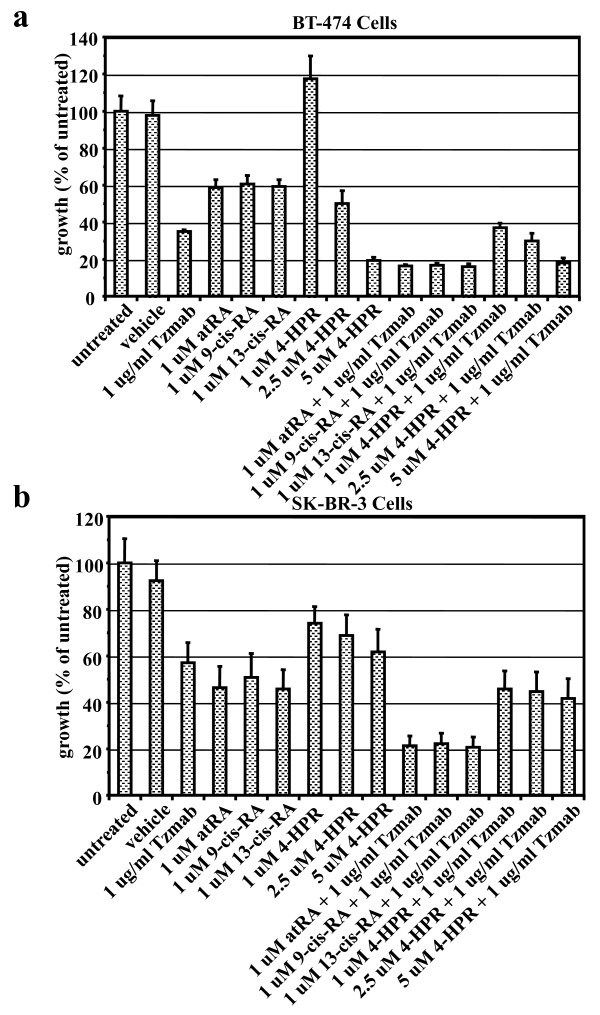
**WST-1 proliferation assay of BT474 cells and SKBR3 cells treated with various retinoid combinations**. WST-1 proliferation assay of **(a) **BT474 cells or **(b) **SKBR3 cells either untreated or treated for 6 days with vehicle (ethanol + PBS), trastuzumab (Tzmab), various retinoids, or their combinations. Results from WST-1 assays are expressed as a percentage of untreated cells. Each value is the mean of three independent experiments (with eight replicate wells for each treatment) ± standard error. atRA, all-*trans *retinoic acid; 4-HPR, *N*-(4-hydroxyphenyl) retinamide (fenretinide); RA, retinoic acid.

HER2-overexpressing ER-positive/BT474 cells or ER-negative/SKBR3 cells were treated with each of the following single agents: 1 μg/ml trastuzumab, 1 μM atRA, 1 μM 9-*cis*-RA, 1 μM 13-*cis*-RA, 1 μM 4-HPR, 2.5 μM 4-HPR, or 5 μM 4-HPR - or with trastuzumab/retinoid combinations. On day 6 following treatment, the effects of the single agents and drug combinations on BT474 or SKBR3 cell growth were examined by the WST-1 proliferation assay. The results from WST-1 assays were expressed as a percentage of untreated cells. The combinations of trastuzumab with the various retinoids showed greater growth inhibition than the single agents alone in both BT474 cells (Figure 4a) and SKBR3 cells (Figure 4b) - with the exception of 4-HPR, which showed minimal ability to enhance trastuzumab-mediated growth inhibition in both cell lines.

### Effect of other retinoids with trastuzumab on cell cycle of ER-positive and ER-negative cells

The cell cycle distribution of BT474 cells or SKBR3 cells was analyzed on day 2 following treatment with single agents or combinations of the drugs. Each drug individually is known to cause G_1 _cell cycle accumulation.

**Figure 5:**
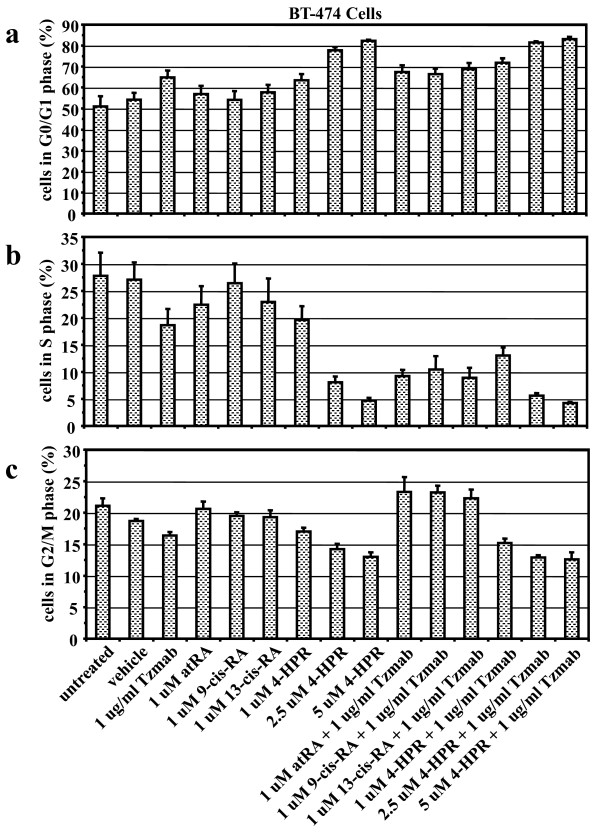
**Cell cycle analysis of BT474 cells treated with various retinoid combinations**. Cell cycle analysis of BT474 cells either untreated or treated for 2 days with vehicle (ethanol + PBS), trastuzumab (Tzmab), various retinoids, or their combinations. On day 2 following treatment, both floating and adherent cells were collected and fixed. Percentages of cells in the **(a) **G_0_/G_1 _phase, **(b) **S phase, and **(c) **G_2_/M phase of the cell cycle were determined by flow cytometric analyses. Results are mean of three independent experiments ± standard error. atRA, all-*trans *retinoic acid; 4-HPR, *N*-(4-hydroxyphenyl) retinamide (fenretinide); RA, retinoic acid.

In BT474 cells, compared with untreated cells and vehicle-treated cells, the single agents trastuzumab and 4-HPR, and the combinations of trastuzumab with the various retinoids, led to an enhanced accumulation of cells in the G_0_/G_1 _phase coupled with a reduction of cells in the S phase of the cell cycle (Figure 5). In general, the drug combinations produced a lower percentage of cells in the S phase than did single agents alone in BT474 cells (Figure 5).

**Figure 6:**
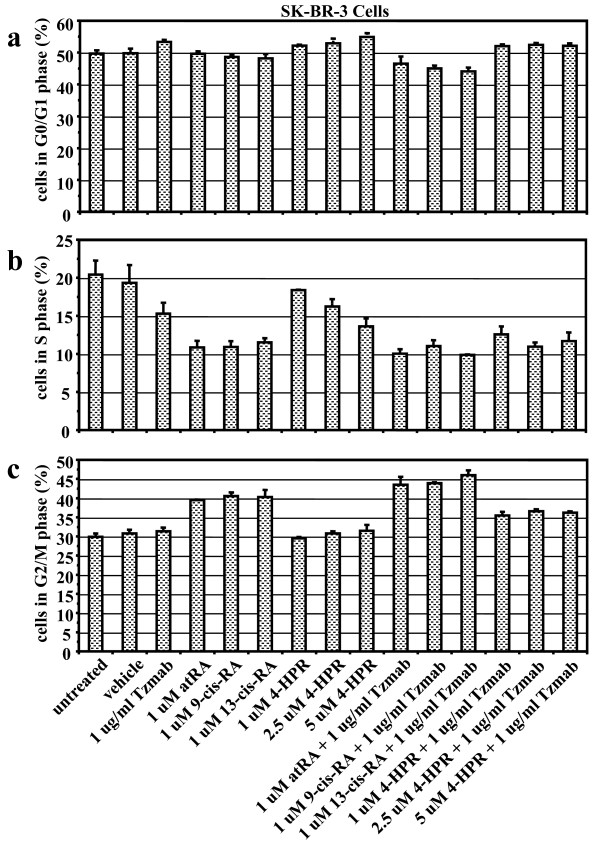
**Cell cycle analysis of SKBR3 cells treated with various retinoid combinations**. Cell cycle analysis of SKBR3 cells either untreated or treated for 2 days with vehicle (ethanol + PBS), trastuzumab (Tzmab), various retinoids, or their combinations. On day 2 following treatment, both floating and adherent cells were collected and fixed. Percentages of cells in the **(a) **G_0_/G_1 _phase, **(b) **S phase, and **(c) **G_2_/M phase of the cell cycle were determined by flow cytometric analyses. Results are mean of three independent experiments ± standard error. atRA, all-*trans *retinoic acid; 4-HPR, *N*-(4-hydroxyphenyl) retinamide (fenretinide); RA, retinoic acid.

In SKBR3 cells, all single agents resulted in a reduced S phase (4-HPR required higher concentrations), although there was only a modest effect of adding trastuzumab to 4-HPR and no additional effect of adding trastuzumab to the other retinoids (Figure 5). Unlike the BT474 cells, none of the drugs or their combinations had a significant impact on the G_1 _phase in SKBR3 cells; rather, the retinoids (excluding 4-HPR) caused an increase in the G_2_/M phase, which was just slightly enhanced by addition of trastuzumab to any of the retinoids (Figure 6).

### Effect of other retinoids with trastuzumab on differentiation of ER-positive and ER-negative cells

**Figure 7:**
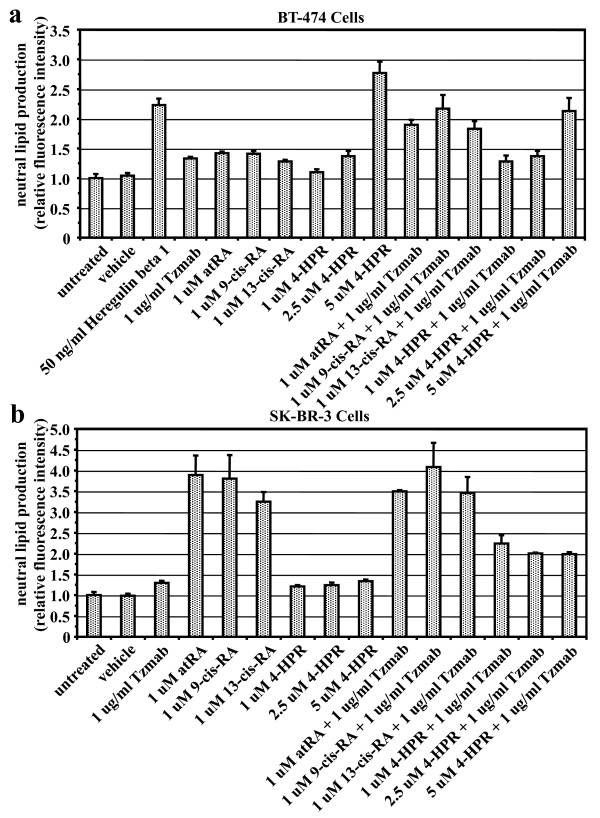
**Differentiation of BT474 cells or SKBR3 cells treated with various retinoid combinations, determined by Nile red staining of neutral lipids**. **(a) **BT474 cells or **(b) **SKBR3 cells were either untreated or treated with vehicle (ethanol + PBS), heregulin β1, trastuzumab (Tzmab), various retinoids, or their combinations. On day 6 following treatment, the cells were collected and stained with the fluorescent dye Nile red. Fluorescence intensities of the cells were analyzed by flow cytometry and expressed relative to the intensity of the untreated cells. Results are mean of three independent experiments ± standard error. atRA, all-*trans *retinoic acid; 4-HPR, *N*-(4-hydroxyphenyl) retinamide (fenretinide); RA, retinoic acid.

Differentiation of BT474 cells or SKBR3 cells was determined by Nile red fluorescent dye staining of neutral lipids on day 6 following treatment with HRGβ1 (positive control), single agents, or combinations of the drugs. In BT474 cells, trastuzumab and single retinoid agents only slightly increased neutral lipid production, although it was significantly enhanced by adding trastuzumab to retinoids (other than 4-HPR) (Figure 7). In SKBR3 cells, single-agent retinoids induced significantly greater neutral lipid production, with the exception of 4-HPR; addition of trastuzumab to retinoids, however, did not enhance the effect of the retinoids, except for 4-HPR (Figure 7).

### Effect of other retinoids with trastuzumab on apoptosis of ER-positive and ER-negative cells

**Figure 8:**
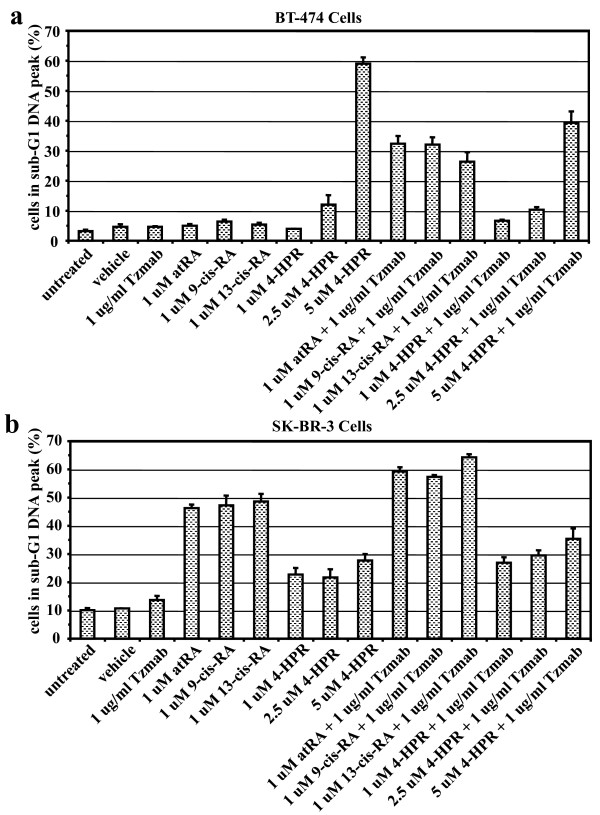
**Detection of apoptotic BT474 cells or SKBR3 cells treated with various retinoid combinations, determined by sub-G**_**1 **_**DNA peak analysis**. **(a) **BT474 cells or **(b) **SKBR3 cells were either untreated or treated with vehicle (ethanol + PBS), trastuzumab (Tzmab), various retinoids, or their combinations. On day 6 following treatment, both floating and adherent cells were collected and examined by sub-G_1 _DNA peak analysis. Percentages of cells in the sub-G_1 _DNA peak were determined by flow cytometry. Results are mean of three independent experiments ± standard error. atRA, all-*trans *retinoic acid; 4-HPR, *N*-(4-hydroxyphenyl) retinamide (fenretinide); RA, retinoic acid.

To detect apoptotic cells, both floating and adherent BT474 or SKBR3 cells were examined by sub-G_1 _DNA peak analysis following treatment for 6 days with single agents or combinations of the drugs. In BT474 cells, sub-G_1 _DNA peak analysis demonstrated that only the combinations of trastuzumab with retinoids induced apoptosis; none of the single agents (except 4-HPR at higher concentrations) induced apoptosis; and 4-HPR alone induced apoptosis at concentrations higher than 1 μM (2.5 μm or 5 μm), and this was not enhanced by trastuzumab (Figure 8). In contrast, the single retinoid agents did cause apoptosis in SKBR3 cells, with 4-HPR having a much weaker effect compared with the other retinoids; the addition of trastuzumab to the retinoids produced a small enhancement in the induction of apoptosis (Figure 8).

### Effect of drugs on receptor signaling

**Figure 9:**
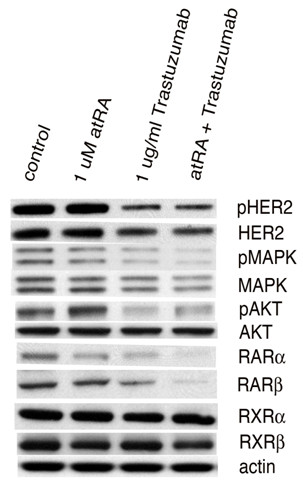
**Effect of treatment of BT474 cells on receptor signaling**. Effect of all-*trans *retinoic acid (atRA), trastuzumab or the combination on HER2 expression level and activity, on AKT expression level and activity, on mitogen-activated protein kinase (MAPK) expression level and activity, and on expression levels of retinoic acid receptor (RAR)α, RARβ, retinoid X receptor (RXR)α and RXRβ in BT474 cells. Drugs used at indicated concentrations. pHER2, pMAPK and pAKT, phospho-HER2, phospho-MAPK and phospho-AKT, respectively.

The effect on receptor signaling of treatment of BT474 cells with atRA, trastuzumab, or both was examined (Figure 9). Single-agent trastuzumab at 1 μg/ml resulted in a moderate decrease in total levels of HER2, and, as expected, a more significant decrease in HER2 activity as reflected by the level of HER2 autophosphorylation. Treatment with atRA at 1 μM had no effect on HER2 expression level or the degree of phosphorylation, and, when added to 1 μg/ml trastuzumab, did not have a significant effect on HER2 expression level or activity. Single-agent atRA also did not significantly affect AKT or MAPK expression or activity. Trastuzumab treatment resulted in partial inhibition of AKT and MAPK activity; while addition of atRA to trastuzumab had no further effect on AKT activity, the combination did appear to result in a small further decrement in MAPK activity.

Single-agent atRA at 1 μM caused a small decrease in the level of RARα (Figure 9). Trastuzumab at 1 μg/ml had a similar effect, and the combination resulted in the greatest decrease in expression level of this receptor. Treatment with atRA did not appear to affect levels of RARβ, RXRα or RXRβ; however, trastuzumab caused a small decrement in expression of RARβ that was enhanced when combined with atRA (Figure 9).

**Figure 10:**
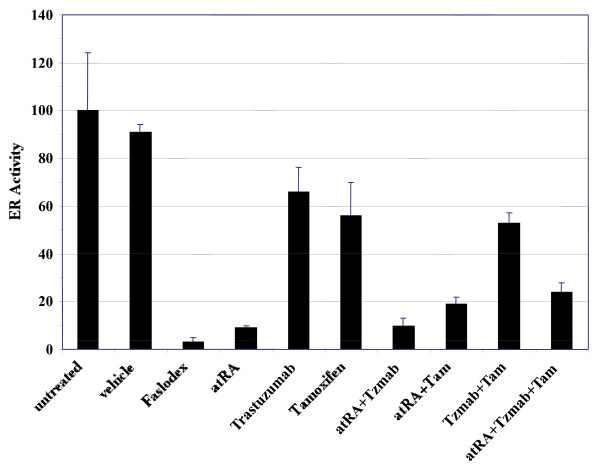
**Estrogen receptor transcriptional activity in BT474 cells**. Estrogen receptor (ER) transcriptional activity in BT474 cells transiently co-transfected with 3× ERE-TATA luciferase and CMV-Renilla luciferase vectors following treatment with 1 μM Faslodex, 1 μM all-*trans *retinoic acid (atRA), 1 μg/ml trastuzumab (Tzmab), 1 μM tamoxifen (Tam), or their combinations. The firefly luciferase activities of the treated cells have been normalized to their Renilla luciferase activities and are expressed as a percentage of activity of untreated cells. Results are mean of three independent experiments ± standard error.

ER transcriptional activity was examined using an ERE assay in BT474 cells. Treatment with single-agent atRA caused a profound inhibition of ERE activity, comparable with the ER downregulator Faslodex (Figure 10). Single-agent trastuzumab caused partial inhibition of ERE activity comparable with that of tamoxifen, implicating peptide growth factor signaling pathway-driven ER activation in these cells; however, adding trastuzumab to tamoxifen did not further inhibit ERE activity. Adding trastuzumab, tamoxifen or both to atRA could not further inhibit ERE activity beyond that of atRA treatment alone.

### Effect of atRA on growth of trastuzumab-resistant BT474 cells

**Figure 11:**
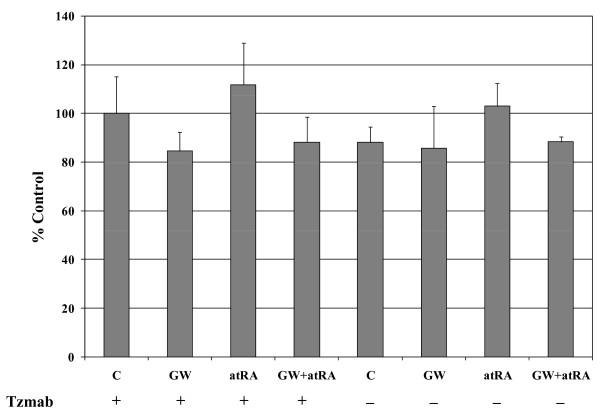
**Effect of atRA on growth of trastuzumab-resistant BT474 cells**. WST-1 proliferation assay of trastuzumab-resistant BT474 cells treated for 6 days with 10 μM GW2974, 1 μM all-*trans *retinoic acid (atRA), or their combination, in the presence or absence of trastuzumab (Tzmab) in the culture media. Each bar is normalized to the growth of control (C, vehicle-treated) cells in trastuzumab-containing media. Each value is the mean of three independent experiments (with three replicate wells for each treatment) ± standard error.

Given the ability of the atRA/trastuzumab combination to synergistically induce apoptosis in BT474 cells under conditions where neither agent alone could do so, it was of interest to examine the activity of atRA in trastuzumab-resistant BT474 cells. Resistance was induced in these cells by long-term culture in trastuzumab-containing media. As shown in Figure 11, atRA did not affect the growth of trastuzumab-resistant BT474 cells, whether used in the presence or absence of trastuzumab in the media. In fact, removal of trastuzumab from the media did not affect growth of these cells. Slight growth inhibition was observed when trastuzumab-resistant cells were treated with 10 μM of the epidermal growth factor receptor/HER2 dual tyrosine kinase inhibitor laptinib analog GW2974 in the presence of trastuzumab-containing media, although this required doses much greater than those typically required to inhibit growth of trastuzumab-sensitive cells; the addition of atRA to GW2974 was not able to enhance growth inhibition observed with the latter agent alone.

## Discussion

HER2 and ER play critical roles in breast cancer and are validated therapeutic targets in this disease. Retinoids have also been shown to inhibit breast cancer growth. We have demonstrated that combining atRA with trastuzumab, tamoxifen, or both results in strong synergistic growth inhibition of BT474 human breast cancer cells. To elucidate the molecular mechanisms underlying this synergistic growth inhibition, we examined the effects of single agents and various drug combinations on cell cycle, differentiation, and apoptosis. We found that treatment with the atRA/trastuzumab and atRA/trastuzumab/tamoxifen combinations caused induction of apoptosis, which was not observed for single drugs or the trastuzumab/tamoxifen or atRA/tamoxifen combinations.

Since we observed that combining atRA with trastuzumab uniquely resulted in apoptosis, we examined the effects of other retinoids with trastuzumab, in both ER-positive (BT474) and ER-negative (SKBR3) HER2-overexpressing human breast cancer cells. We found in BT474 cells that, while none of the single agents (except 4-HPR) induce apoptosis, the combinations of various retinoids with trastuzumab also result in apoptosis. In contrast, the single-agent retinoids (other than 4-HPR) do induce apoptosis in SKBR3 cells (weakest for 4-HPR), and adding trastuzumab to the retinoids causes only a small enhancement of that effect. The pan-retinoid receptor agonists 9-*cis*-RA and 13-*cis*-RA hence behave similarly to atRA, while 4-HPR has a different activity profile. A recent study reported synergistic growth inhibition and induction of apoptosis for the combination of trastuzumab and 9-*cis*-RA in hepatocellular cells [59], suggesting application to a broader range of malignancies; that report demonstrated that trastuzumab inhibited phosphorylation of RXRα and enhanced 9-*cis*-RA-induced RA response element and retinoid X response element activity.

The single agents employed in our study have been reported previously to promote accumulation of cells in the G_0_/G_1 _phase of the cell cycle [36,60-65]. Our results confirm these findings. Compared with untreated cells and vehicle-treated cells, the combinations of trastuzumab with various retinoids lead to enhanced accumulation of BT474 cells in the G_0_/G_1 _phase and SKBR3 cells in the G_2_/M phase, coupled with a reduction of cells in the S phase. We further demonstrate that the combinations generally lead to a greater reduction of cells in the S phase of the cell cycle than the respective single agents in BT474 cells.

Retinoids have also been demonstrated to modulate breast cancer cell growth through differentiation as well as apoptosis [34,35], and to cooperate with heregulin to induce morphologic differentiation (branching morphogenesis) in three-dimensional culture [66]. We have found that the single-agent retinoids, trastuzumab and tamoxifen, individually induce differentiation but not apoptosis in BT474 cells. The combinations of various retinoids and trastuzumab result in greater differentiation than respective single agents in BT474 cells. Compared with untreated cells and vehicle-treated cells, the single retinoid agents alone induce greater differentiation and greater apoptosis in SKBR3 cells than in BT474 cells. The combinations of various retinoids and trastuzumab result in greater apoptosis than, but similar differentiation as, respective single agents alone in SKBR3 cells.

The combinations of trastuzumab and various retinoids do induce apoptosis in both BT474 cells and SKBR3 cells. Our findings are consistent with previous studies that show sensitivity to atRA is decreased in HER2-overexpressing breast cancer cells [41,42]. Consequently, we find that targeting of HER2 by trastuzumab in the presence of retinoids induces apoptosis and greater differentiation in BT474 cells; the potentiation of retinoid-induced apoptosis by trastuzumab was modest in SKBR3 cells. The capacities of retinoids to induce differentiation and apoptosis are thus enhanced when trastuzumab inhibits signaling by HER2. Through the induction of apoptosis, greater differentiation, and effects on cell cycle, the combinations of trastuzumab and various retinoids resulted in greater growth inhibition than single agents alone in both BT474 cells and SKBR3 cells.

Numerous previous studies have suggested promise for the combination of retinoids with anti-estrogens. Tamoxifen was found to potentiate the effect of atRA to inhibit estrogen-induced growth of MCF7 cells [31]. In a rat carcinogen-induced mammary tumor model, the rexinoid bexarotene (Targretin) was able to induce complete remission of the majority of established tumors, and its combination with tamoxifen was more effective than either alone [67]. In this model, there was also some evidence that adding the retinoid to tamoxifen after the development of tamoxifen resistance may restore some sensitivity to tamoxifen, since response rates were higher than when tamoxifen was discontinued and Targretin was used instead [68].

A number of clinical trials have explored the therapeutic potential of retinoids in breast cancer patients or as prevention agents. Fenretinide, Targretin, 9-*cis*-RA, 13-*cis*-RA and atRA have been examined in clinical trials. A small phase II trial of 13-*cis*-RA in 18 heavily pretreated (chemotherapy and endocrine therapy refractory) advanced breast cancer patients yielded no objective responses [69]. Fenretinide had relatively mild and reversible toxicity in a small phase II trial in patients with advanced disease but also showed no clinical activity [70]. A small phase II trial of atRA in patients with hormone refractory metastatic breast cancer showed it to be relatively well tolerated, but noted only one partial response among 14 evaluable patients - although there was marked interpatient variability in pharmacokinetics [71]. A large phase III secondary prevention trial using fenretinide for 5 years after surgical treatment for ductal carcinoma *in situ *or stage I breast cancer revealed no statistically significant effect on the prevention of second contralateral or ipsilateral breast malignancies in the group as a whole, or in distant metastases or survival - although intriguingly there was a reduction of contralateral and ipsilateral breast cancer among premenopausal patients in the study [72], which may suggest a specific interaction with estrogen signaling; at 15-year follow up, the study continued to show the same trend [73].

Given the disappointing results for retinoids as single agents in advanced disease, combinations with other agents become of interest. The combinations of tamoxifen with atRA, retinyl acetate, 9-*cis*-RA, fenretinide, Targretin and retinyl palmitate have been studied in clinical trials.

A pilot breast cancer chemoprevention trial using fenretinide in combination with tamoxifen is being conducted [74]. This agent is also being tested as secondary chemoprevention (of contralateral breast cancer) as a single agent by the Milan group. In patients with advanced disease in a small phase I trial of the 9-*cis*-RA/tamoxifen combination, the dose-limiting toxicities were headache, hypercalcemia and noncardiogenic pulmonary edema; and of nine assessable patients, there was one partial response and one complete response, both in patients who had ER-positive tumors and previous tamoxifen therapy [75]. A phase I/II trial of tamoxifen with or without fenretinide in ER-positive or PR-positive, previously untreated metastatic breast cancer revealed no significant toxicity and improvement or stabilization of disease in 12 of 15 patients [76]. In a phase I/II trial of the atRA/tamoxifen combination in patients with potentially hormone-responsive advanced disease, the dose-limiting toxicity was headache and dermatologic toxicity; and two out of seven patients with measurable disease responded, while seven out of 18 patients with nonmeasurable but evaluable disease had stable disease [77].

Targretin has been tested in patients with metastatic breast cancer, as monotherapy and in combination with tamoxifen for tamoxifen-resistant patients; however, response rates were low - on the order of 3 to 6%, although up to 20% of patients had some clinical benefit [78]. In a phase II study of tamoxifen plus high-dose retinyl acetate in postmenopausal patients with advanced breast cancer, toxicity was generally mild and an overall response rate of 38.5% was reported [79].

In a phase II trial of the fenretinide/tamoxifen combination specifically in advanced disease patients with ER-negative tumors or patients with ER-positive tumors previously treated with tamoxifen, no objective responses were observed although three patients had prolonged stable disease [80]. A recent report with a 2 × 2 trial design found that either low-dose tamoxifen or fenretinide could lower risk of breast neoplasms compared with placebo, but curiously their combination could not, suggesting potential antagonism, although the study was underpowered to detect true differences [81]. A pilot phase II study of IFNβ/retinyl palmitate/tamoxifen in patients with advanced disease showed a clinical response rate of 55% [82]. No reported studies have evaluated the therapeutic effects of a retinoid/trastuzumab combination in a clinical trial, and our results suggest such a strategy could be of benefit.

## Conclusions

In summary, the combinations of various retinoids with trastuzumab, tamoxifen, or both shows strong synergistic inhibition of proliferation accompanied by cell-cycle delay, differentiation, and, for retinoid/trastuzumab combinations, apoptosis in both ER-positive and ER-negative human breast cancer cells. The retinoid/trastuzumab combination resulted in enhanced inhibition of MAPK signaling and downregulation of RARα and RARβ. Treatment with a retinoid and simultaneous inhibition of HER2 and/or ER signaling may thus hold promise as therapy for breast cancer patients.

## Abbreviations

atRA: all-*trans *retinoic acid; CI: combination index; ER: estrogen receptor; EtOH: ethanol; FBS: fetal bovine serum; 4-HPR: *N*-(4-hydroxyphenyl) retinamide (fenretinide); HRGβ1: heregulin β1 (neuregulin); IFN: interferon; MAPK: mitogen-activated protein kinase; PBS: phosphate-buffered saline; RA: retinoic acid; RAR: retinoic acid receptor; RXR: retinoid X receptor; ERE: estrogen response element.

## Competing interests

DCK, MN, LNH and CZ declare that they have no competing interests. MPD has received royalties from DAKO and NeoMarkers, and has served in consulting/advisory board roles for Genentech and NovoNordisk.

## Authors' contributions

MPD conceived of the study, participated in its design, and helped to draft the manuscript. DCK and CZ conducted the experiments; DCK primarily drafted the manuscript, and CZ drafted sections of the manuscript. MN and LNH developed the trastuzumab-resistant BT474 cell line. All authors read and approved the final manuscript.

## References

[B1] DiGiovanna MP DeVita VT Jr, Hellman S, Rosenberg SA Clinical significance of HER-2/*neu *overexpression: Part I Principles and Practice of Oncology 1999 9 Cedar Knolls: Lippincott Williams & Wilkins [Rosenberg SA (Series Editor): *Principles and Practice of Oncology Updates*, vol. 13].

[B2] DiGiovanna MP DeVita VT Jr, Hellman S, Rosenberg SA Clinical significance of HER-2/*neu *overexpression: Part II Principles and Practice of Oncology 1999 10 Cedar Knolls: Lippincott Williams & Wilkins [Rosenberg SA (Series Editor): *Principles and Practice of Oncology Updates*, vol. 13].

[B3] Carter P Presta L Gorman CM Ridgway JBB Henner D Wong WLT Rowland AM Kotts C Carver ME Shepard HM Humanization of an anti-p185^HER2 ^antibody for human cancer therapy Proc Natl Acad Sci USA 1992 89 4285 4289 10.1073/pnas.89.10.4285 1350088 PMC49066

[B4] Chazin VR Kaleko M Miller AD Slamon DJ Transformation mediated by the human *HER-2 *gene independent of the epidermal growth factor receptor Oncogene 1992 7 1859 1866 1354348

[B5] Hudziak RM Lewis GD Winget M Fendly BM Shepard HM Ullrich A p185^*HER2 *^monoclonal antibody has antiproliferative effects in vitro and sensitizes human breast tumor cells to tumor necrosis factor Mol Cell Biol 1989 9 1165 1172 2566907 10.1128/mcb.9.3.1165-1172.1989 PMC362707

[B6] Baselga J Tripathy D Mendelsohn J Baughman S Benz CC Dantis L Sklarin NT Seidman AD Hudis CA Moore J Rosen PP Twaddell T Henderson IC Norton L Phase II study of weekly intravenous recombinant humanized anti-p185^HER2 ^monoclonal antibody in patients with HER2/*neu*-overexpressing metastatic breast cancer J Clin Oncol 1996 14 737 744 8622019 10.1200/JCO.1996.14.3.737

[B7] Cobleigh MA Vogel CL Tripathy D Robert NJ Scholl S Fehrenbacher L Wolter JM Paton V Shak S Lieberman G Slamon DJ Multinational study of the efficacy and safety of humanized anti-HER2 monoclonal antibody in women who have HER2-overexpressing metastatic breast cancer that has progressed after chemotherapy for metastatic disease J Clin Oncol 1999 17 2639 2648 10561337 10.1200/JCO.1999.17.9.2639

[B8] Vogel CL Cobleigh MA Tripathy D Gutheil JC Harris LN Fehrenbacher L Slamon DJ Murphy M Novotny WF Burchmore M Shak S Steward SJ Press M Efficacy and safety of trastuzumab as a single agent in first-line treatment of *HER2*-overexpressing metastatic breast cancer J Clin Oncol 2002 20 719 726 10.1200/JCO.20.3.719 11821453

[B9] Burstein HJ Kuter I Campos SM Gelman RS Tribou L Parker LM Manola J Younger J Matulonis U Bunnell CA Partridge AH Richardson PG Clarke K Shulman LN Winer EP Clinical activity of trastuzumab and vinorelbine in women with HER2-overexpressing metastatic breast cancer J Clin Oncol 2001 19 2722 2730 11352965 10.1200/JCO.2001.19.10.2722

[B10] Pegram MD Lipton A Hayes DF Weber BL Baselga JM Tripathy D Baly D Baughman SA Twaddell T Glaspy JA Slamon DJ Phase II study of receptor-enhanced chemosensitivity using recombinant humanized anti-p185^HER2/^^*neu *^monoclonal antibody plus cisplatin in patients with HER2/*neu*-overexpressing metastatic breast cancer refractory to chemotherapy treatment J Clin Oncol 1998 16 2659 2671 9704716 10.1200/JCO.1998.16.8.2659

[B11] Slamon DJ Leyland-Jones B Shak S Fuchs H Paton V Bajamonde A Fleming T Eiermann W Wolter J Pegram M Baselga J Norton L Use of chemotherapy plus a monoclonal antibody against HER2 for metastatic breast cancer that overexpresses HER2 N Engl J Med 2001 344 783 792 10.1056/NEJM200103153441101 11248153

[B12] Burstein HJ Harris LN Marcom PK Lambert-Falls R Havlin K Overmoyer B Friedlander RJ Gargiulo J Strenger R Vogel CL Ryan PD Ellis MJ Nunes RA Bunnell CA Campos SM Hallor M Gelman R Winer EP Trastuzumab and vinorelbine as first-line therapy for HER2-overexpressin metastatic breast cancer: multicenter phase II trial with clinical outcomes, analysis of serum tumor markers as predictive factors, and cardiac surveillance algorithm J Clin Oncol 2003 21 2889 2895 10.1200/JCO.2003.02.018 12885806

[B13] Seidman AD Fornier MN Esteva FJ Tan L Kaptain S Bach A Panageas KS Arroyo CD Valero V Currie V Gilewski T Theodoulou M Moynahan ME Moasser MM Sklarin N Dicler M D'Andrea G Cristofanilli M Rivera E Hortobagyi GN Norton L Hudis CA Weekly trastuzumab and paclitaxel therapy for metastatic breast cancer with analysis of efficacy by HER2 immunophenotype and gene amplification J Clin Oncol 2001 19 2587 2595 11352950 10.1200/JCO.2001.19.10.2587

[B14] Leyland-Jones B Gelmon K Ayoub JP Arnold A Verma S Dias R Ghahramani P Pharmacokinetics, safety, and efficacy of trastuzumab administered every three weeks in combination with paclitaxel J Clin Oncol 2003 21 3965 3971 10.1200/JCO.2003.12.109 14507946

[B15] Esteva FJ Valero V Booser D Guerra LT Murray JL Pusztai L Cristofanilli M Arun B Esmaeli B Fritsche HA Sneige N L ST Hortobagyi GN Phase II study of weekly docetaxel and trastuzumab for patients with HER-2-overexpressing metastatic breast cancer J Clin Oncol 2002 20 1800 1808 10.1200/JCO.2002.07.058 11919237

[B16] Tedesco KL Thor AD Johnson DH Shyr Y Blum KA Goldstein LJ Gradishar WJ Nicholson BP Merkel DE Murrey D Edgerton SM Sledge GW Docetaxel combined with trastuzumab is an active regimen in HER2 3+overexpressing and fluorescent in situ hybridization-positive metastatic breast cancer: a multi-institutional phase II trial J Clin Oncol 2004 22 1071 1077 10.1200/JCO.2004.10.046 15020608

[B17] Montemurro F Choa G Faggiuolo R Donadio M Minischetti M Durando A Capaldi A Vietti-Ramus G Alabiso O Aglietta M A phase II study of three-weekly docetaxel and weekly trastuzumab in HER2-overexpressing advance breast cancer Oncology 2004 66 38 45 10.1159/000076333 15031597

[B18] Romond EH Perez EA Bryant J Suman VJ Geyer CE Jr Davidson NE Tan-Chiu E Martino S Paik S Kaufman PA Swain SM Pisansky TM Fehrenbacher L Kutteh LA Vogel VG Visscher DW Yothers G Jenkins RB Brown AM Dakhil SR Mamounas EP Lingle WL Klein PM Ingle JN Norman W Trastuzumab plus adjuvant chemotherapy for operable HER2-positive breast cancer N Engl J Med 2005 353 1673 1684 10.1056/NEJMoa052122 16236738

[B19] Piccart-Gebhart MJ Proctor M Leyland-Jones B Goldhirsch A Untch M Smith I Gianni L Baselga J Bell R Jackisch C Cameron D Dowsett M Barrios CH Steger G Huang CS Andersson M Inbar M Lichinitser M Láng I Nitz U Iwata H Thomssen C Lohrisch C Suter TM Rüschoff J Sütö T Greatorex V Ward C Straehle C McFadden E Trastuzumab after adjuvant chemotherapy in HER2-positive breast cancer N Engl J Med 2005 353 1659 1672 10.1056/NEJMoa052306 16236737

[B20] Slamon D Eiermann W Robert N Pienkowski T Martin M Pawlicki M Chan A Smylie M Liu M Falkson C Pinter T Fornander T Shiftan T Valero V Mackey J Tabah-Fisch I Buyse M Lindsay MA Riva A Bee V Pegram M Press M Crown J BCIRG 006: 2nd interim analysis phase III randomized trial comparing doxorubicin and cyclophosphamide followed by docetaxel (AC/T) with doxorubicin and cyclophosphamide followed by docetaxel and trastuzumab (AC/TH) with docetaxel, carboplatin and trastuzumab (TCH) in Her2neu positive early breast cancer patients [abstract 52] Presented at San Antonio Breast Cancer Symposium 2006: December 14 2006; San Antonio

[B21] Dickson RB Lippman ME Control of human breast cancer by estrogen, growth factors, and oncogenes Cancer Treat Res 1988 40 119 165 2908648 10.1007/978-1-4613-1733-3_6

[B22] Jacquemier JD Hassoun J Torrente M Martin PM Distribution of estrogen and progesterone receptors in healthy tissue adjacent to breast lesions at various stages - immunohistochemical study of 107 cases Breast Cancer Res Treat 1990 15 109 117 10.1007/BF01810783 2322649

[B23] Pfahl M Vertebrate receptors: molecular biology, dimerization and response elements Semin Cell Biol 1994 5 95 103 10.1006/scel.1994.1013 8068887

[B24] Mangelsdorf DJ Evans RM The RXR heterodimers and orphan receptors Cell 1995 83 841 850 10.1016/0092-8674(95)90200-7 8521508

[B25] Chambon P A decade of molecular biology of retinoic acid receptors Faseb J 1996 10 940 954 8801176

[B26] Mangelsdorf DJ Umesono K Evans RM Sporn MB, Roberts AB, Goodman DS The retinoid receptors The Retinoids: Biology, Chemistry, and Medicine 1994 2 New York: Raven Press Ltd 319 349

[B27] Simeone AM Tari AM How retinoids regulate breast cancer cell proliferation and apoptosis Cell Mol Life Sci 2004 61 1475 1484 10.1007/s00018-004-4002-6 15197471 PMC11138852

[B28] Chen AC Guo X Derguini F Gudas LJ Human breast cancer cells and normal mammary epithelial cells: retinol metabolism and growth inhibition by the retinol metabolite 4-oxoretinol Cancer Res 1997 57 4642 4651 9377581

[B29] Butler WB Fontana JA Responses to retinoic acid of tamoxifen-sensitive and -resistant sublines of human breast cancer cell line MCF-7 Cancer Res 1992 52 6164 6167 1423259

[B30] Rubin M Fenig E Rosenauer A Menendez-Botet C Achkar C Bentel JM Yahalom J Mendelsohn J Miller WH Jr 9-Cis retinoic acid inhibits growth of breast cancer cells and down-regulates estrogen receptor RNA and protein Cancer Res 1994 54 6549 6556 7987855

[B31] Demirpence E Balaguer P Trousse F Nicolas JC Pons M Gagne D Antiestrogenic effects of all-trans-retinoic acid and 1,25-dihydroxyvitamin D_3 _in breast cancer cells occur at the estrogen response element level but through different molecular mechanisms Cancer Res 1994 54 1458 1464 8137248

[B32] Rousseau C Pettersson F Couture MC Paquin A Galipeau J Mader S Miller WH Jr The N-terminal of the estrogen receptor (ERα) mediates transcriptional cross-talk with the retinoic acid receptor in human breast cancer cells J Steroid Biochem Mol Biol 2003 86 1 14 10.1016/S0960-0760(03)00255-3 12943740

[B33] Schneider SM Offterdinger M Huber H Grunt TW Activation of retinoic acid receptor alpha is sufficient for full induction of retinoid responses in SK-BR-3 and T47D human breast cancer cells Cancer Res 2000 60 5479 5487 11034091

[B34] Grunt TW Dittrich E Offterdinger M Schneider SM Dittrich C Huber H Effects of retinoic acid and fenretinide on the c-erbB-2 expression, growth and cisplatin sensitivity of breast cancer cells Br J Cancer 1998 78 79 87 9662255 10.1038/bjc.1998.446 PMC2062943

[B35] Bacus SS Kiguchi K Chin D King CR Huberman E Differentiation of cultured human breast cancer cells (AU-565 and MCF-7) associated with loss of cell surface *HER-2*/*neu *antigen Mol Carcinog 1990 3 350 362 10.1002/mc.2940030607 1980588

[B36] Offterdinger M Schneider SM Huber H Grunt TW Retinoids control the expression of c-ErbB receptors in breast cancer cells Biochem Biophys Res Comm 1998 251 907 913 10.1006/bbrc.1998.9570 9791009

[B37] Offterdinger M Schneider SM Huber H Grunt TW Expression of c-erbB-4/HER4 is regulated in T47D breast carcinoma cells by retinoids and vitamin D3 Biochem Biophys Res Commun 1999 258 559 564 10.1016/S0006-291X(00)90001-9 10383375

[B38] Schneider SM Offterdinger M Huber H Grunt TW Involvement of nuclear steroid/thyroid/retinoid receptors and of protein kinases in the regulation of growth and of c-erbB and retinoic acid receptor expression in MCF-7 breast cancer cells Breast Cancer Res Treat 1999 58 171 181 10.1023/A:1006377006816 10674883

[B39] Pellegrini R Mariotti A Tagliabue E Bressan R Bunone G Coradini D Della Valle G Formelli F Cleris L Radice P Modulation of markers associated with tumor aggressiveness in human breast cancer cell lines by *N*-(4-hydroxyphenyl) retinamide Cell Growth Diff 1995 6 863 869 7547508

[B40] Flicker SH Schneider SM Offterdinger M Dittrich E Fazeny B Valenta R Huber H Dittrich C Grunt TW Tyrosine kinase signaling pathways control the expression of retinoic acid receptor-alpha in SK-BR-3 breast cancer cells Cancer Lett 1997 115 63 72 10.1016/S0304-3835(97)04715-0 9097980

[B41] Siwak DR Mendoza-Gamboa E Tari AM HER2/neu uses Akt to suppress retinoic acid response element binding activity in MDA-MB-453 breast cancer cells Int J Oncol 2003 23 1739 1745 14612949

[B42] Tari AM Lim SJ Hung MC Esteva FJ Lopez-Berestein G Her2/neu induces all-trans retinoic acid (ATRA) resistance in breast cancer cells Oncogene 2002 21 5224 5232 10.1038/sj.onc.1205660 12149644

[B43] Keith WN Douglas F Wishart GC McCallum HM George WD Kaye SB Brown R Co-amplification of erbB2, topoisomerase II alpha and retinoic acid receptor alpha genes in breast cancer and allelic loss at topoisomerase I on chromosome 20 Eur J Cancer 1993 29A 1469 1475 10.1016/0959-8049(93)90022-8 8104440

[B44] Rao GN Ney E Herbert RA Changes associated with delay of mammary cancer by retinoid analogues in transgenic mice bearing *c-neu *oncogene Breast Cancer Res Treat 1999 58 241 254 10.1023/A:1006315716713 10718486

[B45] Wu KD Zhang Y Xu XC Hill J Celestino J Kim HT Mohsin SK Hilsenbeck SG Lamph WW Bissonette R Brown PH The retinoid X receptor-selective retinoid, LGD1069, prevents the development of estrogen receptor-negative mammary tumors in transgenic mice Cancer Res 2002 62 6376 6380 12438218

[B46] Liby K Rendi M Suh N Royce DB Risingsong R Williams CR Lamph W Labrie F Krajewski S Xu X Kim H Brown P Sporn MB The combination of the rexinoid, LG100268, and a selective estrogen receptor modulator, either arzoxifene or acolbifene, synergizes in the prevention and treatment of mammary tumors in an estrogen receptor-negative model of breast cancer Clin Cancer Res 2006 12 5902 5909 10.1158/1078-0432.CCR-06-1119 17020999

[B47] Lasfargues EY Coutinho WG Redfield ES Isolation of two human tumor epithelial cell lines from solid breast carcinomas J Natl Cancer Inst 1978 61 967 978 212572

[B48] Kallioniemi OP Kallioniemi A Kurisu W Thor A Chen LC Smith HS Waldman FM Pinkel D Gray JW *ERBB2 *amplification in breast cancer analyzed by fluorescence *in situ *hybridization Proc Natl Acad Sci USA 1992 89 5321 5325 10.1073/pnas.89.12.5321 1351679 PMC49283

[B49] Lewis GD Figari I Fendly B Wong WL Carter P Gorman C Shepard HM Differential responses of human tumor cell lines to anti-p185^HER2 ^monoclonal antibodies Cancer Immunol Immunother 1993 37 255 263 10.1007/BF01518520 8102322 PMC11038979

[B50] Grunt TW Saceda M Martin MB Lupu R Dittrich E Krupitza G Harant H Huber H Dittrich C Bidirectional interactions between the estrogen receptor and the c-erbB-2 signaling pathways: heregulin inhibits estrogenic effects in breast cancer cells Int J Cancer 1995 63 560 567 10.1002/ijc.2910630417 7591267

[B51] Chou TC Talalay P Quantitative analysis of dose-effect relationships: the combined effects of multiple drugs or enzyme inhibitors Adv Enzyme Regul 1984 22 27 55 10.1016/0065-2571(84)90007-4 6382953

[B52] Chou TC Hayball MP CalcuSyn: Windows Software for Dose Effect Analysis 1996

[B53] Chou TC Motzer RJ Tong Y Bosl GJ Computerized quantitation of synergism and antagonism of taxol, topotecan, and cisplatin against human teratocarcinoma cell growth: a rational approach to clinical protocol design J Natl Cancer Inst 1994 86 1517 1524 10.1093/jnci/86.20.1517 7932806

[B54] Crissman HA Hirons GT Staining of DNA in live and fixed cells Methods Cell Biol 1994 41 195 209 full_text 7532262 10.1016/s0091-679x(08)61718-5

[B55] Rodes JF Berreur-Bonnenfant J Tremolieres A Brown SC Modulation of membrane fluidity and lipidic metabolism in transformed rat fibroblasts induced by the sesquiterpenic hormone farnesylacetone Cytometry 1995 19 217 225 10.1002/cyto.990190305 7736867

[B56] Wang CX Koay DC Edwards A Lu Z Mor G Ocal IT DiGiovanna MP In vitro and in vivo effects of combination of trastuzumab (Herceptin) and tamoxifen in breast cancer Breast Cancer Res Treat 2005 92 251 263 10.1007/s10549-005-3375-z 16155796

[B57] Zhang JX Labaree DC Mor G Hochberg RB Estrogen to antiestrogen with a single methylene group resulting in an unusual steroidal estrogen receptor modulator J Clin Endocrinol Metab 2004 89 3527 3535 10.1210/jc.2003-032005 15240642

[B58] Narayan M Wilken JA Harris LN Baron AT Kimbler KD Maihle NJ Trastuzumab-induced HER reprogramming in 'resistant' breast carcinoma cells Cancer Res 2009 69 2191 2194 10.1158/0008-5472.CAN-08-1056 19276389

[B59] Tatebe H Shimizu M Shirakami Y Tsurumi H Moriwaki H Synergistic growth inhibition by 9-cis-retinoic acid plus trastuzumab in human hepatocellular carcinoma cells Clin Cancer Res 2008 14 2806 2812 10.1158/1078-0432.CCR-07-4708 18451248

[B60] Osborne CK Boldt DH Clark GM Trent JM Effects of tamoxifen on human breast cancer cell cycle kinetics: accumulation of cells in early G_1 _phase Cancer Res 1983 43 3583 3585 6861130

[B61] Sutherland RL Green MD Hall RE Reddel RR Taylor IW Tamoxifen induces accumulation of MCF 7 human mammary carcinoma cells in the G_0_/G_1 _phase of the cell cycle Eur J Cancer Clin Oncol 1983 19 615 621 10.1016/0277-5379(83)90177-3 6683633

[B62] Lane HA Beuvink I Motoyama AB Daly JM Neve RM Hynes NE ErbB2 potentiates breast tumor proliferation through modulation of p27^Kip1^-Cdk2 complex formation: receptor overexpression does not determine growth dependency Mol Cell Biol 2000 20 3210 3223 10.1128/MCB.20.9.3210-3223.2000 10757805 PMC85615

[B63] Pegram M Hsu S Lewis G Pietras R Beryt M Sliwkowski M Coombs D Baly D Kabbinavar F Slamon D Inhibitory effects of combinations of HER-2/*neu *antibody and chemotherapeutic agents used for treatment of human breast cancers Oncogene 1999 18 2241 2251 10.1038/sj.onc.1202526 10327070

[B64] Sliwkowski MX Lofgren JA Lewis GD Hotaling TE Fendly BM Fox J Nonclinical studies addressing the mechanism of action of trastuzumab (Herceptin) Semin Oncol 1999 26 Suppl 12 60 70 10482195

[B65] Argiris A Wang CX Whalen SG DiGiovanna MP Synergistic interactions between tamoxifen and trastuzumab (Herceptin) Clin Cancer Res 2004 10 1409 1420 10.1158/1078-0432.CCR-1060-02 14977844

[B66] Offterdinger M Schneider SM Grunt TW Heregulin and retinoids synergistically induce branching morphogenesis of breast cancer cells cultivated in 3D collagen gels J Cell Physiol 2003 195 260 275 10.1002/jcp.10237 12652653

[B67] Bischoff ED Gottardis MM Moon TE Heyman RA Lamph WW Beyond tamoxifen: the retinoid X receptor-selective ligand LGD1069 (TARGRETIN) causes complete regression of mammary carcinoma Cancer Res 1998 58 479 484 9458093

[B68] Bischoff ED Heyman RA Lamph WW Effect of the retinoid X receptor-selective ligand LGD1069 on mammary carcinoma after tamoxifen failure J Natl Cancer Inst 1999 91 2118 10.1093/jnci/91.24.2118 10601384

[B69] Cassidy J Lippman M Lacroix A Peck G Phase II trial of 13-cis-retinoic acid in metastatic breast cancer Eur J Cancer Clin Oncol 1982 18 925 928 10.1016/0277-5379(82)90239-5 6962067

[B70] Modiano MR Dalton WS Lippman SM Joffe L Booth AR Meyskens FL Jr Phase II study of fenretinide (*N*-[4-hydroxyphenyl]retinamide) in advanced breast cancer and melanoma Invest New Drugs 1990 8 317 319 10.1007/BF00171846 2148744

[B71] Sutton LM Warmuth MA Petros WP Winer EP Pharmacokinetics and clinical impact of all-trans retinoic acid in metastatic breast cancer: a phase II trial Cancer Chemother Pharmacol 1997 40 335 341 10.1007/s002800050666 9225952

[B72] Veronesi U De Palo G Marubini E Costa A Formelli F Mariani L Decensi A Camerini T Del Turco MR Di Mauro MG Muraca MG Del Vecchio M Pinto C D'Aiuto G Boni C Campa T Magni A Miceli R Perloff M Malone WF Sporn MB Randomized trial of fenretinide to prevent second breast malignancy in women with early breast cancer J Natl Cancer Inst 1999 91 1847 1856 10.1093/jnci/91.21.1847 10547391

[B73] Veronesi U Mariani L Decensi A Formelli F Camerini T Miceli R Di Mauro MG Costa A Marubini E Sporn MB De Palo G Fifteen-year results of a randomized phase III trial of fenretinide to prevent second breast cancer Ann Oncol 2006 17 1065 1071 10.1093/annonc/mdl047 16675486

[B74] Conley B O'Shaughnessy J Prindiville S Lawrence J Chow C Jones E Merino MJ Kaiser-Kupfer MI Caruso RC Podgor M Goldspiel B Venzon D Danforth D Wu S Noone M Goldstein J Cowan KH Zujewski J Pilot trial of the safety, tolerability, and retinoid levels of *N*-(4-hydroxyphenyl) retinamide in combination with tamoxifen in patients at high risk for developing invasive breast cancer J Clin Oncol 2000 18 275 283 10637240 10.1200/JCO.2000.18.2.275

[B75] Lawrence JA Adamson PC Caruso R Chow C Kleiner D Murphy RF Venzon DJ Shovlin M Noone M Merino M Cowan KH Kaiser M O'Shaughnessy J Zujewski J Phase I clinical trial of alitretinoin and tamoxifen in breast cancer patients: toxicity, pharmacokinetic, and biomarker evaluations J Clin Oncol 2001 19 2754 2763 11352969 10.1200/JCO.2001.19.10.2754

[B76] Cobleigh MA Dowlatshahi K Deutsch TA Mehta RG Moon RC Minn F Benson AB Rademaker AW Ashenhurst JB Wade JL Phase I/II trial of tamoxifen with or without fenretinide, an analog of vitamin A, in women with metastatic breast cancer J Clin Oncol 1993 11 474 477 8445423 10.1200/JCO.1993.11.3.474

[B77] Budd GT Adamson PC Gupta M Homayoun P Sandstrom SK Murphy RF McLain D Tuason L Peereboom D Bukowski RM Ganapathi R Phase I/II trial of all-trans retinoic acid and tamoxifen in patients with advanced breast cancer Clin Cancer Res 1998 4 635 642 9533531

[B78] Esteva FJ Glaspy J Baidas S Laufman L Hutchins L Dicler M Tripathy D Cohen R DeMichele A Yocum RC Osborne CK Hayes DF Hortobagyi GN Winer E Demetri GD Multicenter phase II study of oral bexarotene for patients with metastatic breast cancer J Clin Oncol 2003 21 999 1006 10.1200/JCO.2003.05.068 12637463

[B79] Boccardo F Canobbio L Resasco M Decensi AU Pastorino G Brema F Phase II study of tamoxifen and high-dose retinyl acetate in patients with advanced breast cancer J Cancer Res Clin Oncol 1990 116 503 506 10.1007/BF01613002 2229142 PMC12201906

[B80] Zujewski J Pai L Wakefield L Giusti R Dorr FA Flanders C Caruso R Kaiser M Goodman L Merino M Gossard M Noone MA Denicoff A Venzon D Cowan KH O'Shaughnessy JA Tamoxifen and fenretinide in women with metastatic breast cancer Breast Cancer Res Treat 1999 57 277 283 10.1023/A:1006216409688 10617304

[B81] Decensi A Robertson C Guerrieri-Gonzaga A Serrano D Cazzaniga M Mora S Gulisano M Johansson H Galimberti V Cassano E Moroni SM Formelli F Lien EA Pelosi G Johnson KA Bonanni B Randomized double-blind 2 × 2 trial of low-dose tamoxifen and fenretinide for breast cancer prevention in high-risk women J Clin Oncol 2009 27 3749 3756 10.1200/JCO.2008.19.3797 19597031 PMC2799048

[B82] Recchia F Sica G de Filippis S Discepoli S Rea S Torchio P Frati L Interferon-beta, retinoids, and tamoxifen in the treatment of metastatic breast cancer: a phase II study J Interferon Cytokine Res 1995 15 605 610 10.1089/jir.1995.15.605 7553230

